# Repurposing anti-inflammatory drugs for fighting planktonic and biofilm growth. New carbazole derivatives based on the NSAID carprofen: synthesis, *in silico* and *in vitro* bioevaluation

**DOI:** 10.3389/fcimb.2023.1181516

**Published:** 2023-08-23

**Authors:** Florea Dumitrascu, Mino R. Caira, Speranta Avram, Catalin Buiu, Ana Maria Udrea, Ilinca Margareta Vlad, Irina Zarafu, Petre Ioniță, Diana Camelia Nuță, Marcela Popa, Mariana-Carmen Chifiriuc, Carmen Limban

**Affiliations:** ^1^ ”C. D. Nenitzescu” Institute of Organic and Supramolecular Chemistry, Center for Organic Chemistry, Bucharest, Romania; ^2^ Department of Chemistry, University of Cape Town, Cape Town, South Africa; ^3^ Department of Anatomy, Animal Physiology, and Biophysics, Faculty of Biology, University of Bucharest, Bucharest, Romania; ^4^ Department of Automatic Control and Systems Engineering, Politehnica University of Bucharest, Bucharest, Romania; ^5^ Laser Department, National Institute for Laser, Plasma and Radiation Physics, Magurele, Romania; ^6^ Research Institute of the University of Bucharest—ICUB, University of Bucharest, Bucharest, Romania; ^7^ Department of Pharmaceutical Chemistry, Faculty of Pharmacy, “Carol Davila” University of Medicine and Pharmacy, Bucharest, Romania; ^8^ Department of Organic Chemistry, Biochemistry and Catalysis, Faculty of Chemistry, University of Bucharest, Bucharest, Romania; ^9^ Department of Botany and Microbiology, University of Bucharest, Bucharest, Romania; ^10^ Biological Sciences Section, Romanian Academy, Bucharest, Romania

**Keywords:** carprofen, antibacterial, antibiofilm, ESKAPE pathogens, new carbazole derivatives

## Abstract

**Introduction:**

One of the promising leads for the rapid discovery of alternative antimicrobial agents is to repurpose other drugs, such as nonsteroidal anti-inflammatory agents (NSAIDs) for fighting bacterial infections and antimicrobial resistance.

**Methods:**

A series of new carbazole derivatives based on the readily available anti-inflammatory drug carprofen has been obtained by nitration, halogenation and N-alkylation of carprofen and its esters. The structures of these carbazole compounds were assigned by NMR and IR spectroscopy. Regioselective electrophilic substitution by nitration and halogenation at the carbazole ring was assigned from H NMR spectra. The single crystal X-ray structures of two representative derivatives obtained by dibromination of carprofen, were also determined. The total antioxidant capacity (TAC) was measured using the DPPH method. The antimicrobial activity assay was performed using quantitative methods, allowing establishment of the minimal inhibitory/bactericidal/biofilm eradication concentrations (MIC/MBC/MBEC) on Gram-positive (*Staphylococcus aureus, Enterococcus faecalis*) and Gram-negative (*Escherichia coli, Pseudomonas aeruginosa*) strains. Computational assays have been performed to assess the drug- and lead-likeness, pharmacokinetics (ADME-Tox) and pharmacogenomics profiles.

**Results and discussion:**

The crystal X-ray structures of 3,8-dibromocarprofen and its methyl ester have revealed significant differences in their supramolecular assemblies. The most active antioxidant compound was **1i**, bearing one chlorine and two bromine atoms, as well as the CO2Me group. Among the tested derivatives, **1h** bearing one chlorine and two bromine atoms has exhibited the widest antibacterial spectrum and the most intensive inhibitory activity, especially against the Gram-positive strains, in planktonic and biofilm growth state. The compounds **1a** (bearing one chlorine, one NO2 and one CO2Me group) and **1i** (bearing one chlorine, two bromine atoms and a CO2Me group) exhibited the best antibiofilm activity in the case of the *P. aeruginosa* strain. Moreover, these compounds comply with the drug-likeness rules, have good oral bioavailability and are not carcinogenic or mutagenic. The results demonstrate that these new carbazole derivatives have a molecular profile which deserves to be explored further for the development of novel antibacterial and antibiofilm agents.

## 1 Introduction

The emergence of bacterial infections and of antimicrobial resistance raises global concerns and requires urgent action for the development of novel effective antibacterial strategies. Among diverse bacterial species involved in human and animal infections, four of them are listed as the most dangerous because of their resistance mechanisms, i.e., *Staphylococcus aureus (S. aureus*) and *Enterococcus faecalis (E. faecalis)* among Gram-positive and *Escherichia coli* (*E. coli)* and *Pseudomonas aeruginosa* (*P. aeruginosa)* from the Gram-negative species. These species are among the most important opportunistic pathogens, being involved in a wide range of hospital- and community-acquired infections, exhibiting resistance to many of the currently used antibiotics, including last resort ones, such as colistin, and often harboring a multi-drug resistance profile (Sivick and Mobley, 2010; Sharma G. et al., 2014; Lazaris et al., 2017; Martinez et al., 2018; Vestergaard et al., 2019; Guo et al., 2020; Ewers et al., 2022; Laborda et al., 2022).

MDR clones, such methicillin-resistant *S. aureus* (MRSA) strains, vancomycin-resistant enterococci (VRE), producing severe infections mainly in immunocompromised patients and *P. aeruginosa* strains, which are responsible for almost 10% of all hospital-acquired infections worldwide, are now reported globally with high prevalence, raising public health concerns, and fostering the research for better understanding of the molecular basis of their virulence and resistance to developing efficient drugs or vaccines (Ruoff et al., 1990; Arias and Murray, 2012; Chatterjee et al., 2016; Schulte and Munson, 2019; Cascioferro et al., 2021). In these bacteria, antibiotic resistance genes are often associated with mobile genetic elements, which facilitate their transmission and dissemination, under the antibiotic selective pressure, present not only in the clinical settings, but also in the natural environment, as already demonstrated for wastewater (Tansirichaiya et al., 2021; Malekian et al., 2022). Moreover, these species exhibit high biofilm development ability, leading to chronic colonization, as well as to recalcitrant and relapsing infections, thus urgently requiring the rapid discovery and development of alternative therapeutic strategies (Linden, 2002; Foxman, 2010; Subashchandrabose and Mobley, 2017; Pang et al., 2019; Venkateswaran et al., 2022).

If in the case of the Gram-positive bacteria, novel antimicrobial agents (quinupristin/dalfopristin and linezolid) have been introduced as alternatives to fight MRSA and VRE infections, for the Gram-negative resistant pathogens no promising lead is available. Moreover, resistance to these new compounds has already been reported (Humphries et al., 2012; Miller et al., 2013). It has been shown that *E. faecalis* exhibits a robust and rapid adaptation capacity to stressors, requiring the development of new “antievolution” strategies and targets (Olar et al., 2022).

Bacterial biofilms, defined as monospecific or multi-specific bacterial communities adhered to an inert substratum or viable tissues and protected by a self-secreted extracellular polymeric matrix are highly tolerant to diverse stressors, including drugs, biocides or host defense effectors, exhibiting the so-called phenotypic resistance to antibacterials which can be up to thousands of times higher than the level of resistance in planktonic cells (Wu et al., 2019). A new generation of antibacterial strategies is directed towards developing anti-biofilm strategies, acting on different levels of biofilm development, without interfering with the microbial growth, being therefore called anti-virulence and exhibiting a low selective pressure for resistance (Lewis, 2008; Yin et al., 2014; Michiels et al., 2016; Fisher et al., 2017; Chang et al., 2020).

Recent studies highlight the role of nonsteroidal anti-inflammatory drugs (NSAIDs) as potential sources of novel antibacterial agents (Salih et al., 2016; Chan et al., 2017).

Carbazole derivatives have attracted considerable attention in medicinal chemistry because they exhibit a broad spectrum of pharmacological and biological activities, including antimicrobial (Borger et al., 2017), antitubercular (Guillonneau et al., 2005), antitumour (Hieda et al., 2014), antioxidant (Grande et al., 2021) beta-adrenergic blocking (Dineshkumar et al., 2011), antidiabetic (Bandgar et al., 2012) and anti-inflammatory (Cuong et al., 2008) properties. The carbazole moiety is found in alkaloids extracted from the taxonomically related higher plants of the genus *Clausena*, *Glycosmis*, and *Murraya*. Thus, the carbazole ring is present in natural medicinally active substances, such as murrayafoline A with antifungal activity (Is et al., 2010). Carbazole derivatives are also used in the materials science field, as optoelectronic materials, conducting polymers, and synthetic dyes (Martin and Prasad, 2006; Srinivas et al., 2011).

**Scheme 1:**
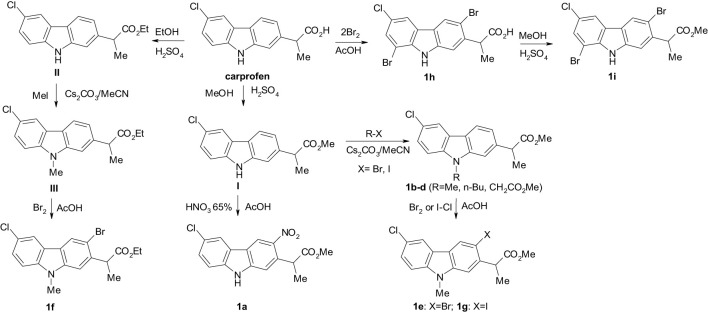
The synthesis of new carbazole derivatives starting from the NSAID carprofen.

Carprofen, (*RS*)-2-(6-chloro-9*H*-carbazol-2-yl)propanoic acid (**Scheme 1**), is a NSAID of the propionic acid class. The expensive starting materials, stringent reaction conditions and the lengthy synthetic steps, made the development of other NSAIDs more attractive and the use of carprofen in humans stopped after about ten years, on commercial grounds, presently remaining available to veterinarians for prescribing as a supportive treatment.

**Figure 1:**
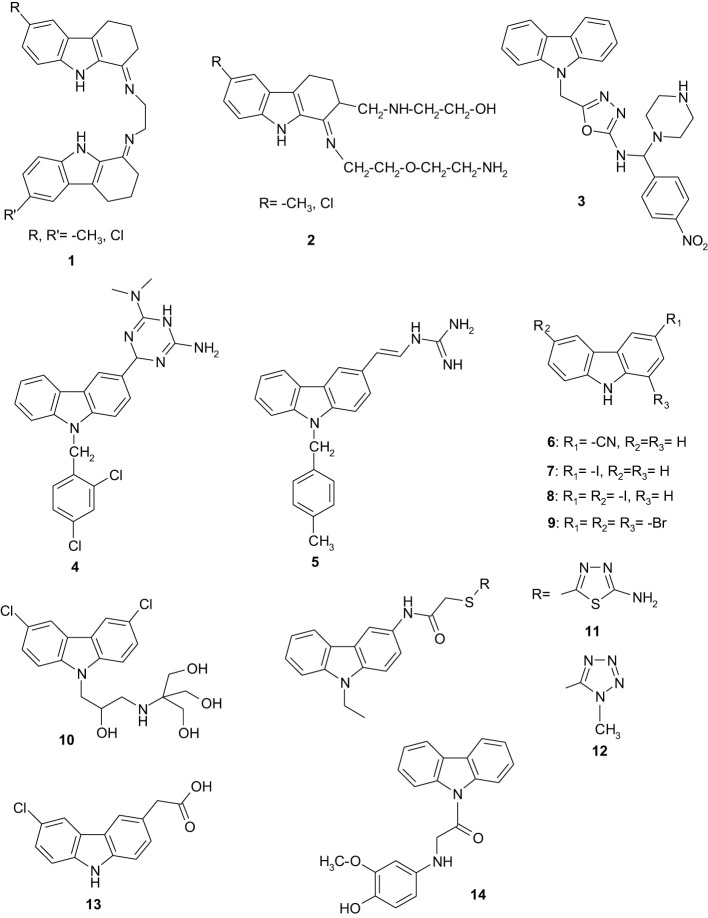
The chemical structure of the compounds **1**- **14**.

Despite this, the carprofen scaffold still remains a source for new effective and safe analgesic and anti-inflammatory drugs, but many studies report its antimicrobial and antiviral activity. The Mannich reaction of 1-oxo-1,2,3,4-tetrahydrocarbazoles with paraformaldehyde and ethylenediamine or ethanolamine yielded N,N’-bis(1,2,3,4-tetrahydrocarbazol-1-ylidene)ethane-1,2-diamines or 2-{[1-(2-(2-aminoethoxy)ethylimino)-1,2,3,4-tetrahydrocarbazol-2-yl-methyl]amino}ethanols which are new compounds that have been screened for antibacterial and antifungal activities. The compounds having substituents at the C-6 position were found to exhibit pronounced antimicrobial activities (**Figure 1**- compounds 1 and 2) (Sharma D. et al., 2014). From a series of novel 5-[(*9H*-carbazol-9-yl)methyl]-N-[(substituted phenyl)(piperazin-1-yl)methyl]-1,3,4-oxadiazol-2-amines evaluated for their antibacterial, antifungal and anticancer activities, the 5-[(9*H*-carbazol-9-yl)methyl]-N-[(4-nitrophenyl)(piperazin-1-yl)methyl]-1,3,4-oxadiazol-2-amine exhibited the best antibacterial and antifungal activity (**Figure 1**- compound 3) (Tansirichaiya et al., 2021). Xue and co-workers synthesized and evaluated the antibacterial and antifungal activities of novel carbazole derivatives containing an aminoguanidine, dihydrotriazine, thiosemicarbazide, semicarbazide or isonicotinic moiety (Xue et al., 2021). Among them, 6-(9-(2,4-dichlorobenzyl)-9*H*-carbazol-3-yl)-N^2^,N^2^-dimethyl-3,6-dihydro-1,3,5-triazine-2,4-diamine (**Figure 1**- compound 4) showed the highest potential as a therapeutic agent, with a MIC value of 0.5–2 µg/mL against the tested bacterial strains. The (*E*)-2-((9-(4-methylbenzyl)-9*H*-carbazol-3-yl)methylene)hydrazine-1-carboximidamide (**Figure 1**- compound 5) also exhibited strong antibacterial activity and docking simulation suggested that binding to dihydrofolate reductase might account for the antimicrobial activity of the compounds. Dabrovolskas and co-workers synthesized carbazole-based compounds containing halogens, cyano and alkyl groups, and their antibacterial activity was evaluated using a disk diffusion method. The antioxidant activity was evaluated using free 1,1-diphenyl-2-picryl-hydrazyl radical scavenging assay and ferric reducing antioxidant power methods. 3-Cyano-9*H*-carbazole (**Figure 1**- compound 6), 3-iodo-9*H*-carbazole (**Figure 1**- compound 7) and 3,6-diiodo-9*H*-carbazole (**Figure 1**- compound 8) showed a stronger antibacterial activity against *Bacillus subtilis* compared to amoxicillin as reference drug. 1,3,6-Tribromo-9*H*-carbazole (**Figure 1**- compound 9) showed a stronger activity against *Escherichia coli*. All tested compounds showed a weak to moderate antioxidant activity by the stated assay methods (Dabrovolskas et al., 2020).

Carbazole derivatives exhibit several mechanisms of antibacterial action. One of these mechanisms consists in increasing the membrane permeability by inhibiting specific enzymatic processes. Membrane-active compounds are a promising solution for treating persistent infections. Thus, 2-((3-(3,6-dichloro-*9H*-carbazol-9-yl)-2-hydroxypropyl)amino)-2-(hydroxymethyl)propane-1,3-diol (**Figure 1**- compound 10) that reduces the transmembrane potential of Gram-positive and Gram-negative bacteria and causes mislocalization of essential membrane-associated proteins, provides new opportunities for the development of potent broad-spectrum antimicrobial agents (Eun et al., 2012). Another mechanism supports the idea that carbazole derivatives can interact with bacterial DNA replication and repair (Zhang et al., 2018), by inhibiting the DNA polymerase (Foxman, 2010), being reported to inhibit the growth of different Gram-positive and Gram-negative bacteria, such as *B. cereus*, *Staphylococcus aureus, Listeria monocytogenes, Mycobacterium tuberculosis, M. bovis, M. neoaurum, M. smegmatis, Salmonella choleraesuis, Enterobacter aerogenes, Pseudomonas aeruginosa, Klebsiella pneumoniae, E. coli, Helicobacter pylori*) (Lee et al., 2014; Addla et al., 2016; Yimer et al., 2018).

Carbazole derivatives resulting from the reaction of 2-chloro-N-(9-ethyl-9*H*-carbazol-3-yl)acetamide with appropriate mercapto-heterocyclics, i.e. 2-(4-methyl-4*H*-[1,2,4]triazol-3-yl)sulfanyl-N-(9-ethyl-9*H*-carbazol-3-yl)acetamide (**Figure 1**- compound 11) and 2-(1-methyl-1*H*-tetrazol-5-yl)sulfanyl-N-(9-ethyl-9*H*-carbazol-3-yl)acetamide (**Figure 1**- compound 12), showed similar activity to that of ketoconazole against *Candida albicans* NRRL Y- 27077 and *C. glabrata* ATCC 36583 (Shirin et al., 2006).

The anti-inflammatory activity of carprofen can be explained by the fact that it is able to exert a dose-dependent inhibition on neutrophil phagocytosis and particularly on their chemotaxis. This property could have implications in the clinical treatment of tuberculosis (TB) as the primary route of pathogenesis of *M. tuberculosis* necessitates the initial, phagocytic uptake of bacteria by host cells. The action of carprofen on mycobacteria limits the possibility for an emergence of resistant mutants and offers alternative mechanisms of action to the current anti-TB drugs. Also, 2-(6-chloro-9*H*-carbazol-3-yl)acetic acid (**Figure 1**- compound 13), a carprofen analogue, with similar antimycobacterial activity to carprofen, may be the first in a series of novel antimycobacterial carbazoles. Further research is needed for this repurposed drug and its derivatives for human studies (Kaplancıklı, 2014).

A series of carbazole analogues conjugated with different (un)substituted aminophenols were synthesized using as key intermediate 1-(9*H*-carbazol-9-yl)-2-chloroethanone, which was synthesized by N-acylation of carbazole with chloroacetyl chloride. The obtained compounds were studied for their 2,2-diphenyl-1-picrylhydrazyl (DPPH) radical scavenging activity. The coupling of the key intermediate with different aminophenols enhances the radical scavenging activity. 1-(9*H*-Carbazole-9-yl)-2-(4-hydroxy-3-methoxyphenylamino)ethanone (**Figure 1**- compound 14), bearing an electron donating methoxy substituent in the phenolic moiety, showed more potent inhibition of DPPH radical scavenging activity, the latter also being more potent than that displayed by the standard butylated hydroxy anisole. These analogues may be useful in the treatment of pathologies involving free radical oxidation (Maitra et al., 2020).

*In silico* techniques may provide a quick and cost-effective analysis that accurately predicts the pharmacokinetic and pharmacodynamic profiles of antimicrobial compounds (Avram et al., 2005; Naik et al., 2010; Buiu et al., 2016).

Considering the acute need for the rapid development of novel antibiotics, one possible strategy for speeding the process is to repurpose other drugs for fighting bacterial infections and antimicrobial resistance. Thus, in this study we have investigated the antibacterial potential of a series of new carbazole derivatives based on the readily available anti-inflammatory drug carprofen. As the antioxidant activity could directly or indirectly interfere with the antibiotic activity, this feature has also been evaluated for the tested compounds.

## 2 Materials and methods

### 2.1 Chemistry

All chemicals were purchased from Sigma Aldrich (St. Louis, MO, USA), Aladdin Reagent (Shanghai, China). The esters I and II were prepared according to the methods described in previous articles (Avram et al., 2002; Bordei Telehoiu et al., 2020; Liu et al., 2021). ^1^H-and ^13^C-NMR spectra were recorded on a Varian Gemini 300BB spectrometer (Varian, Palo Alto, CA, USA) operating at 300 MHz for ^1^H and 75 MHz for ^13^C. CDCl_3_ CD_3_COCD_3_ or DMSO-d6, were used as solvent and TMS (δ = 0.00 ppm) as an internal standard. Chemical shifts are reported as δ values in ppm as referenced to TMS. Multiplicities are recorded as s (singlet), d (doublet), t (triplet), q (quartet), dd (doublet of doublets), m (multiplet). Coupling constants (J) are expressed in Hz. The IR spectra were recorded on a Fourier-transform (FT)-IR Vertex 70 spectrometer (Bruker Optik GmbH, Ettlingen, Germany). The elemental analysis was performed on a Costech Instruments EAS 32 apparatus (Costech Analytical Technologies, Valencia, CA, USA). Melting points were measured using a Boetius hot plate microscope (Carl Zeiss, Jena, Germany) and were uncorrected.

**Methyl 2-(6-chloro-3-nitro-9*H*-carbazol-2-yl)propionate** (**1a**): to solution obtained by dissolving 1.5 g (5.2 mmol) methyl ester of carprofen (**I**) in 20 mL glacial AcOH at 40-50 °C was added dropwise 0.4 mL (5.2 mmol) nitric acid. The reaction mixture was stirred for 10 h at room temperature and the yellow precipitate was filtered and washed on the filter with water. The compound was crystallized from nitromethane as light-yellow crystals with mp 199-201 °C. Yield 79%. Calcd. for C_16_H_13_ClN_2_O_4_ (332.75) C 57.76, H 3.94, N 8.42; found C 58.11, H 4.21, N 8.66. IR (ATR, solid) 1724 cm^−1^ (CO), 2948 cm^−1^ (CH), 3329 cm^−1^ (NH). ^1^H-NMR (300 MHz, DMSO-d_6_, δ_ppm_, *J*_Hz_): 1.57 (d, 3H, Me, *J* = 7.1 Hz), 3.56 (s, 3H, MeO), 4.44 (q, 1H, CHMe, *J* = 7.1 Hz), 7.45 (dd, 1H, *J* = 8.6, 2.1 Hz, H-7), 7.58 (s, 1H, H-1), 7.57 (d, *J* = 8.6 Hz, 1H, H-8), 8.39 (d, 1H, *J* = 2.1 Hz, H-5), 9.04 (s, 1H, H-4), 12.1 (s, 1H, NH). ^13^C-NMR (75 MHz, DMSO-d_6_, δ_ppm_): 17.7 (Me), 42.6 (CHMe), 51.8 (MeO), 112.2, 113.3, 119.8, 121.0, 127.0 (C-1, C-4, C-5, C-7, C-8), 120.2, 123.5, 124.6, 134.0, 139.6, 140.7, 142.7 (C-2, C-3, C-6, C-4a, C-4b, C-8a, C-9a), 173.4 (CO).

#### 2.1.1 General procedure for *N*-alkylation of carprofen esters (1b-d, III)

To a solution obtained by dissolving of 5 mmol carprofen esters **I** or **II** in 20 mL MeCN were added 20 mmol halogenated compound (MeI, *n*-BuI, BrCH_2_COOMe) and 20 mmol Cs_2_CO_3_. The mixture was heated for 12 h under reflux. After cooling of the reaction mixture, the solid was removed by filtration. The solvent was evaporated to a small volume and under stirring was poured into water. The precipitate was filtered and purified by crystallization from a suitable solvent (Udrea et al., 2018).

**Methyl 2-(6-chloro-9-methyl-9*H*-carbazol-2-yl)propanoate** (**1b**). The compound was crystallized from acetonitrile as colorless crystals with mp 129-131°C. Yield 91%. IR (ATR, solid) 1730 cm^−1^ (CO), 2937, 2975 cm^−1^. ^1^H-NMR (300 MHz, CDCl_3_, δ_ppm_, *J*_Hz_): 1.62 (d, 3H, Me, *J*=7.0 Hz), 3.69 (s, 3H, MeO), 3.80 (s, 3H, NMe), 3.93 (q, 1H, CHMe, *J*=7.0 Hz), 7.18 (dd, 1H, *J*=8.2, 1.4 Hz, H-3), 7.29 (d, 1H, *J*=8.6 Hz, H-8), 7.32 (d, 1H, 1.4 Hz, H-1), 7.39 (dd, 1H, *J*=8.6 Hz, 2.0 Hz, H-7) 7.96 (d, 1H, *J*=8.2 Hz, H-4), 8.21 (s, 1H, *J*=2.0 Hz, H-5).^13^C-NMR (75 MHz, CDCl_3_, δ_ppm_): 19.2 (Me), 29.3 (NMe), 46.2 (CHMe), 52.3 (MeO), 107.6, 109.5, 119.2, 120.0, 120.8, 125.7 (C-1, C-3, C-4, C-5, C-7, C-8), 121.2, 123.8, 124.6, 139.3, 139.7, 141.8 (C-2, C-6, C-4a, C-4b, C-8a, C-9a), 176.0 (COO).

**Methyl 2-[9-(*n*-butyl)-6-chloro-9*H*-carbazol-2-yl]propanoate** (**1c**). The compound was crystallized from benzene as colorless crystals with mp 54-56°C. Yield 67%. Calcd. for C_20_H_22_ClNO_2_ (343.86) C 69.86, H 6.45, N 4.07; Found C 70.11, H 6.61, N 4.33. IR (ATR, solid) 1730 cm^−1^ (CO), 2863, 2925, cm^−1^ (CH).^1^H-NMR (300 MHz, CDCl_3_, δ_ppm_, *J*_Hz_): 0.96 (t, 3H, *J*=7.4 Hz, Me), 1.35-1.43 (m,2H, CH_2_), 1.62 (d, 3H, Me, *J*=7.0 Hz), 1.78-1,87 (m,2H, CH_2_), 3.70 (s, 3H, MeO), 3.93 (q, 1H, CHMe, *J*=7.0 Hz), 4.27 (t, 2H, *J*=7.4 Hz, NCH_2_), 7.18 (dd, 1H, *J*=8.2, 1.4 Hz, H-3), 7.29 (d, 1H, *J*=8.6 Hz, H-8), 7.32 (d, 1H, 1.4 Hz, H-1), 7.39 (dd, 1H, *J*=8.6 Hz, 2.0 Hz, H-7) 7.96 (d, 1H, *J*=8.2 Hz, H-4), 8.21 (s, 1H, *J*=2.0 Hz, H-5).^13^C-NMR (75 MHz, CDCl_3_, δ_ppm_): 14.0 (Me), 19.2 (Me), 20.6, 31.2, 43.0 (3CH_2_), 46.1 (CHMe), 52.3 (MeO), 107.8, 109.5, 119.7, 120.0, 120.7, 125.6 (C-1, C-3, C-4, C-5, C-7, C-8), 121.1, 123.8, 124.4, 139.12, 139.15, 141.2 (C-2, C-6, C-4a, C-4b, C-8a, C-9a), 175.2 (COO).

**Methyl 2-[6-chloro-9-(methoxycarbonylmethyl)-9*H*-carbazol-2-yl]propanoate** (**1d**). The compound was crystallized from methanol as colorless crystals with mp 101-102°C. Yield 68%. Calcd. for C_19_H_18_ClNO_4_ (359.81) C 63.43, H 5.04, N 3.89; Found C 63.72, H 5.35, N 4.12. IR (ATR, solid) 1734 cm^−1^ (CO), 2946 cm^−1^ (CH). ^1^H-NMR (300 MHz, CDCl_3_, δ_ppm_, *J*_Hz_): 1.60 (d, 3H, Me, *J*=7.0 Hz), 3.68, 3.74 (2s, 6H, 2MeO), 3.91 (q, 1H, CHMe, *J*=7.0 Hz), 7.19-7.24 (m, 3H, H-1, H-3, H-8), 7.39 (dd, 1H, *J*=8.6 Hz, 2.0 Hz, H-7), 7.97 (d, 1H, *J*=8.2 Hz, H-4), 8.00 (d, 1H, *J*=2.0 Hz, H-5). ^13^C-NMR (75 MHz, CDCl_3_, δ_ppm_): 19.2 (Me), 44.7 (NCH_2_), 46.1 (CHMe), 52.3, 52.7 (2MeO), 107.6, 109.5, 120.0, 120.3, 121.0, 126.1 (C-1, C-3, C-4, C-5, C-7, C-8), 121.6, 124.3, 125.5, 139.3, 139.7, 141.3 (C-2, C-6, C-4a, C-4b, C-8a, C-9a), 168.7, 175.2 (2COO).

**Ethyl 2-(6-chloro-9-methyl-9*H*-carbazol-2-yl)propanoate (III)**. The compound was crystallized from acetonitrile as colorless crystals with mp 102-104°C. Yield 71%. Calcd. for C_18_H_18_ClNO_2_ (315.80) C 68.46, H 5.75, N 4.44; Found C 68.77, H 5.92, N 4.71. IR (ATR, solid) 1721 cm^−1^ (CO), 2869, 2925, 2970, 3041cm^−1^ (CH).^1^H-NMR (300 MHz, CDCl_3_, δ_ppm_, *J*_Hz_): 1.22 (t, 3H, Me, *J*=7.1 Hz), 1.61 (d, 3H, Me, *J*=7.0 Hz), 3.80 (s, 3H, NMe), 3.92 (q, 1H, CHMe, *J*=7.0 Hz), 4.07-4.25(m, 2H, CH_2_O), 7.18 (dd, 1H, *J*=8.2, 1.6 Hz, H-3), 7.28 (d, 1H, *J*=8.6 Hz, H-8), 7.33 (d, 1H, 1.6 Hz, H-1), 7.38 (dd, 1H, *J*=8.6 Hz, 2.0 Hz, H-7) 7.95 (d, 1H, *J*=8.2 Hz, H-4), 7.99 (s, 1H, *J*=2.0 Hz, H-5).^13^C-NMR (75 MHz, CDCl_3_, δ_ppm_): 14.1,19.2 (2Me), 29.2 (NMe), 46.2 (CHMe), 52.3 (CH_2_O), 107.4, 109.3, 119.1, 119.9, 120.6, 125.6 (C-1, C-3, C-4, C-5, C-7, C-8), 121.0, 123.7, 124.4, 139.4, 139.6, 141.7 (C-2, C-6, C-4a, C-4b, C-8a, C-9a), 174.7 (COO).

#### 2.1.2 Bromination procedure of the esters 1b and III

To a solution of 10 mmol of ester **1b** or **III** dissolved in 30 mL AcOH at 40°C was added dropwise 10 mmol of Br_2_ dissolved in 5mL AcOH and then the reaction mixture was stirred for 2 h. By cooling the reaction mixture, 3-bromocarbazoles **1e** and **1f** were obtained as colourless crystals. From the filtrate, by precipitation with water, a second crop of 3-bromocarbazoles **1e, 1f** was obtained. The esters **1e** and **1f** were purified by crystallization from a suitable solvent.

**Methyl 2-(3-bromo-6-chloro-9-methyl-9*H*-carbazol-2-yl)propanoate** (**1e**). The compound was crystallized from acetonitrile as colorless crystals with mp 177-179 °C. Yield 75%. Calcd. for C_17_H_15_BrClNO_2_ (380.67) C 53.64, H 3.97, N 3.68; Found C 53.89, H 4.27, N 3.86. IR (ATR, solid) 1716 cm^-1^ (CO), 2948, 2983, cm^-1^ (CH). ^1^H-NMR (300 MHz, CDCl_3_, δ_ppm_, *J*_Hz_): 1.59 (d, 3H, Me, *J*=7.0 Hz), 3.71 (s, 3H, MeO), 3.81 (s, 3H, NMe), 4.31 (q, 1H, CHMe, *J*=7.0 Hz), 7.29 (d, 1H, *J*=8.5 Hz, H-8), 7.34 (s, 1H, H-1), 7.45 (dd, 1H, H-7, *J*=8.5, 2.0 Hz, H-7), 7.96 (d, 1H, *J*=2.0 Hz, H-5), 8.21 (s, 1H, H-4). ^13^C-NMR (75 MHz, CDCl_3_, δ_ppm_): 18.1 (Me), 29.3 (NMe), 45.0 (CHMe), 52.2 (MeO), 108.0, 109.6, 120.1, 124.5, 126.4 (C-1, C-4, C-5, C-7, C-8), 114.4 (C-Br), 122.4, 122.5, 124.8, 137.7, 139.8, 140.7 (C-2, C-6, C-4a, C-4b, C-8a, C-9a), 174.9 (COO).

**Ethyl 2-(3-bromo-6-chloro-9-methyl-9*H*-carbazol-2-yl)propanoate** (**1f**). The compound was crystallized from ethanol as colorless crystals with mp 97-98 °C. Yield 71%. Calcd. for C_18_H_17_BrClNO_2_ (394.70) C 54.78, H 4.34, N 3.55; Found C 55.12, H 4.70, N 3.86. IR (ATR, solid) 1716 cm^-1^ (CO), 2948, 2983, cm^-1^ (CH). ^1^H-NMR (300 MHz, CDCl_3_, δ_ppm_, *J*_Hz_): 1.24 (d, 3H, *J*=7.0 Hz, Me), 1.59 (d, 3H, Me, *J*=7.0 Hz), 3.77 (s, 3H, NMe), 4.11-4.27 (m, 2H, CH_2_O), 4.40 (q, 1H, CHMe, *J*=7.0 Hz), 7.24 (d, 1H, *J*=8.5 Hz, H-8), 7.34 (s, 1H, H-1), 7.45 (dd, 1H, H-7, *J*=8.5, 2.0 Hz, H-7), 7.91 (d, 1H, *J*=2.0 Hz, H-5), 8.17 (s, 1H, H-4). ^13^C-NMR (75 MHz, CDCl_3_, δ_ppm_): 14.3 (Me), 18.1 (Me), 29.4 (NMe), 45.4 (CHMe), 61.1 (CH_2_O), 108.2, 109.7, 120.2, 124.6, 126.5 (C-1, C-4, C-5, C-7, C-8), 114.5 (C-Br), 122.5, 122.6, 124.9, 138.0, 139.9, 140.9 (C-2, C-6, C-4a, C-4b, C-8a, C-9a), 174.6 (COO).

**Methyl 2-(3-iodo-6-chloro-9-methyl-9*H*-carbazol-2-yl)propanoate** (**1g**). To the solution obtained by dissolving 1.5 g (5 mmol) of carprofen derivative **1b** in 20 mL glacial AcOH at 40°C was added 7 mmol anhydrous sodium acetate and then 7 mmol iodine monochloride in 5 mL glacial AcOH. The reaction mixture was kept under stirring at 40°C for 4 h. By cooling the reaction mixture, pure 3-iodocarprofen was obtained as colourless crystals after filtration. From the filtrate, by precipitation with water, a second fraction of 3-iodocarbazole **1g** was obtained. The compound was crystallized from ethanol as colorless crystals with mp 151-152 °C. Yield 76%. Calcd. for C_17_H_15_ClINO_2_ (427.67) C 47.74, H 3.54, N 3.82; Found C 52.79, H 3.89, N 4.22. IR (ATR, solid) 1716 cm^-1^ (CO), 2948, 2983, cm^-1^ (CH). ^1^H-NMR (300 MHz, CDCl_3_, δ_ppm_, *J*_Hz_): 1.59 (d, 3H, Me, *J*=7.0 Hz), 3.71 (s, 3H, MeO), 3.81 (s, 3H, NMe), 4.31 (q, 1H, CHMe, *J*=7.0 Hz), 7.29 (d, 1H, *J*=8.5 Hz, H-8), 7.34 (s, 1H, H-1), 7.45 (dd, 1H, H-7, *J*=8.5, 2.0 Hz), 7.96 (d, 1H, *J*=2.0 Hz, H-5), 8.21 (s, 1H, H-4).^13^C-NMR (75 MHz, CDCl_3_, δ_ppm_): 19.0 (Me), 29.3 (NMe), 49.8 (CHMe), 52.2 (MeO), 88.5 (C-I), 107.4, 109.5, 120.1, 124.9, 126.4, 131.3 (C-1, C-4, C-5, C-7, C-8), 122.2, 123.3, 124.9, 139.8, 140.5, 141.7 (C-2, C-6, C-4a, C-4b, C-8a, C-9a), 176.0 (COO).

**2-(3,8-Dibromo-6-chloro-9*H*-carbazol-2-yl)propanoic acid** (**1h**). To the solution obtained by dissolving 1.4 g (5 mmol) carprofen in 20 mL glacial AcOH at 50 °C was added dropwise 10 mmol bromine in 5 mL glacial AcOH. The reaction mixture was kept at 50 °C for 30 min. After cooling of the reaction mixture, the formed precipitate was filtered and washed on the filter with water and cold ethanol. The compound was crystallized from acetonitrile as colorless crystals with mp 237-240°C (dec.). Yield 78%. Calcd. for C_15_H_10_Br_2_ClNO_2_ (431.51) C 41.75, H 2.34, N 3.25; Found C 42.09, H 2.70, N 3.55. IR (ATR, solid) 1721 cm^−1^ (CO), 2869, 2925, 2970, 3041cm^−1^ (CH).^1^H-NMR (300 MHz, DMSO-D_6_, δ_ppm_, *J*_Hz_): 1.43 (d, 3H, Me, *J*=7.1 Hz), 4.16 (q, 1H, CHMe, *J*=7.1 Hz), 7.56 (s, 1H, H-1), 7.67 (d, 1H, *J*=1.8 Hz, H-5), 8.30 (d, 1H, *J*=1.8 Hz, H-7), 8.49 (s, 1H, H-4), 11.66 (s, 1H, NH).^13^C-NMR (300 MHz, DMSO-D_6_): 18.6 (Me), 45.2 (CHMe), 104.5 (C-8, C-Br), 111.0 (C-1), 114.9 (C-3, C-Br), 120.4 (C-5), 124.1(C-4), 128.1 (C-7), 122.9, 124.0, 125.5, 137.9, 140.3 (C-2, C-6, C-4a, C-4b, C-8a, C-9a), 175.2 (COO).

**Methyl 2-(3,8-dibromo-6-chloro-9*H*-carbazol-2-yl)propanoate** (**1i**). To a solution of 5 mmol 3,8-dibromocarprofen dissolved in 25 mL methanol was added dropwise 0.4 ml H_2_SO_4_. The reaction mixture was kept at room temperature for 48 h. The ester precipitates as pure product from the reaction medium. After cooling the reaction mixture, the precipitate was filtered and washed with water on the filter. Resulted colorless crystals from 2-propanol with mp 192–194°C. Yield 82%. Calcd. for C_16_H_12_Br_2_ClNO_2_ (445.54) C 43.13, H 2.71, N 3.14; Found C 43.40, H 3.09, N 3.33; IR (ATR, solid) 1701 cm^−1^ (CO), 3355 cm^−1^ (NH). ^1^H-NMR (300 MHz, CDCl_3_, δ_ppm_, *J*_Hz_): 1.59 (d, 3H, Me, *J*=7.1 Hz), 3.74 (s, 3H, MeO), 4.39 (q, 1H, CHMe, *J*=7.1 Hz), 7.41 (s, 1H, H-1), 7.54 (d, 1H, *J*=1.8 Hz, H-5), 7.79 (dd, 1H, *J*=1.8, 0.6 Hz, H-7), 8.09 (s, 1H, H-4), 8.30 (bs, 1H, NH). ^13^C-NMR (75 MHz, CDCl_3_, δ_ppm_): 18.4 (Me), 45.2 (CHMe), 52.5 (MeO), 104.3 (C-8), 111.0 (C-1), 119.3, 125.1, 128.4 (C-1, C-4, C-5, C-7), 115.6 (C-3), 123.5, 123.8, 125.7, 137.3, 138.6, 139.1 (C-2, C-6, C-4a, C-4b, C-8a, C-9a), 175.1 (COO).

#### 2.1.3 Single-crystal X-ray analyses

Crystals of **1h** and **1i** were mounted on a Bruker Apex II four-circle diffractometer for intensity data-collection at 173 (2) K. Data-reduction and multi-scan absorption corrections were applied to the intensities and the structures were solved by direct methods. Isotropic refinement of the non-hydrogen atoms by full-matrix least-squares methods followed and in the later cycles of refinement the atoms were treated anisotropically. All hydrogen atoms were in different Fourier syntheses. They were subsequently included in idealized positions on their parent atoms and in a riding model. The CIF files are accessible from the Cambridge Structural Database (see below) and contain all details of the above procedures and the structural results, as well as the software used for the computations.

### 2.2 Computational assay

#### 2.2.1 Molecule preparation

3D and Simplified Molecular Input Line Entry (SMILES) files of the compounds **1a**-**1i** (Favia et al., 2012) were obtained by Molecular Operating Environment (MOE) software (Avram et al., 2002).

In addition, the optimization of the minimum energies of the compounds was determined using the MOE software by Forcefield MMFF94x with a gradient of 0.05. After minimization, the Gasteiger partial charges were applied to all compounds.

#### 2.2.2 Assessment of compounds’ drug- and lead-likeness features

To appreciate the features of drug-likeness, the chemical compounds **1a**-**1i** were evaluated by several filters (Salih et al., 2016) such as -Lipinski, Ghose, Veber, and Egan used by Swiss ADME web service (Udrea et al., 2020).

#### 2.2.3 Computational pharmacokinetics (ADME-Tox) and pharmacogenomics profiles

SMILES files of molecules **1a**-**1i** were downloaded into the admetSAR2 database (Vlad et al., 2020) to examine *absorption, distribution, metabolism, and excretion* properties (ADME). From all the pharmacokinetics items available in the admetSAR2 database, we selected Human Intestinal Absorption, Blood-Brain Barrier, P-glycoprotein inhibitor/substrate, Plasma protein binding, and primarily renal uptake transporter (OCT2) inhibitor parameters (Yang et al., 2019).

Detailed predicted toxicity profiles of **1a**-**1i** were obtained by admetSAR2 database. We selected the following toxicity items: eye corrosion/irritation, AMES mutagenesis, hepatotoxicity, respiratory toxicity, mitochondrial toxicity, nephrotoxicity, honeybee toxicity, crustacea aquatic toxicity, and fish aquatic toxicity (Vlaicu et al., 2019).

The pharmacogenomic profile extracted from the admetSAR2 database was used to predict the capacity of the compounds to be substrates or inhibitors of CYP2D6, CYP3A4, CYP1a2, CYP2C19, and CYP2C9 (Udrea et al., 2021).

### 2.3 Antioxidant activity of compounds

The DPPH method is largely used for measuring the antioxidant activity as it is fast, simple and allows comparison with other data from the literature obtained by using a similar approach. Fresh stock solutions of compounds **1a-1i** at the concentration of 2 mg/mL in acetone were prepared, as well as a solution of DPPH in the same solvent with a concentration of 0.2 mg/mL. Ascorbic acid was used as reference. For TAC measurements, to 1 mL of DPPH solution was added 1 mL of stock solution of each compound and the mixture kept in the dark for 30 min, followed by the absorbance measurement at 517 nm, using UVD-3500 UV-Vis double beam spectrophotometer. The TAC percentage was calculated following the equation:


$$Inhibition\;\%=\frac{Abs_{i}-Abs_{\left(30\;min\right)}}{Abs_{i}}\times 100$$

where *Abs_i_* is the initial absorbance of the mixture and the *Abs_30 min_* is the absorbance measured after 30 min (Remes et al., 2012; Paun et al., 2013; Bem et al., 2018).

### 2.4 Microbiological assays

The antimicrobial activity assay was performed on *S. aureus* ATCC 25923, *E. faecalis* ATCC 29212*E. coli* ATCC 25922 and *P. aeruginosa* ATCC 27853, using previously reported quantitative methods, allowing establishment of the minimal inhibitory concentration (MIC) (nutrient broth microdilution method), minimal bactericidal concentration (MBC) (viable cell count on solid media) and minimal biofilm inhibitory concentration (MBIC) (purple violet microtiter method) (Marinas et al., 2015; Yang et al., 2019; Limban et al., 2020). For the broth microdilutions assay, ten two-fold serial dilutions have been performed in 96-well microplates, starting from stock solutions of 10 mg/mL in dimethyl-sulfoxide (DMSO), the achieved concentrations being from 5 to 0.009 mg/mL. The wells containing the respective dilutions have been then seeded with a bacterial inoculum of standard density (i.e., 10^6^ colony forming units/mL) prepared from fresh bacterial culture in sterile saline. Sterility and bacterial cultures controls have been used in the experiment. The microplates were incubated overnight at 37^0^C and then, the MIC was recorded as the lowest concentration that inhibited the visible bacterial growth, the culture medium remaining clear similar to the sterility control. The assay was performed in duplicate, and the results were presented as mean ± standard deviation (SD). For the MBC assay, known volumes from the well corresponding to the MIC value as well as from the previous wells where the microbial growth has been absent and the well content remained clear, a volume of 10 µl has been spotted on agar medium. After overnight incubation, the bacterial growth has been inspected and the MBC values have been recorded as the lowest concentrations of the tested compounds that have completely inhibited the bacterial growth. To evaluate the anti-biofilm features of the new compounds, the same micro-plates used for the MIC assay have been used. Their content has been discarded after reading the MIC values, the wells have been gently washed three times with phosphate buffered saline to remove the unattached bacteria and then, the biofilm developed on the plastic wall was fixed with cold methanol for 5 minutes, stained with 1% purple violet solution for 20 minutes and finally, the colored biofilm has been resuspended in 33% acetic acid solution. The intensity of the blue color has been compared with that of the negative and positive culture controls and the MBIC has been recorded as the lowest concentration that inhibited the biofilm development, as shown by the lack of blue color, similar with the negative control. The assay was performed in duplicate and the results were presented as mean ± SD.

## 3 Results and discussion

### 3.1 Chemistry

New carbazole derivatives **1a-i** and **III** were synthesized by nitration, halogenation, N-alkylation followed by subsequent halogenation, starting from the NSAID carprofen and its esters **I** and **II** (**Scheme 1**). Esters **I** and **II** were obtained by esterification of commercial carprofen with methanol and ethanol in the presence of H_2_SO_4_ (Avram et al., 2002; Favia et al., 2012; Dumitrascu et al., 2022).

The nitration of the methyl ester of carprofen **I** dissolved in glacial acetic acid with HNO_3_ 65% at room temperature gave the 3-nitrocarprofen derivative **1a** in good yield as a yellow solid (**Scheme 1**).

*N*-Alkylation of carprofen esters **I** and **II** was performed with alkyl iodides (R-I, R=Me, *n*-Bu) and methyl bromoacetate by a slightly modified method described by Favia et al (Borger et al., 2017). (**Scheme 1**) to obtain **1b-d** and **III**.

Regioselective reaction of compounds **1b** and **III** with bromine in glacial acetic acid afforded the corresponding 3-bromo derivatives **1e** and **1f** (**Scheme 1**). The iodination of **1b** with iodine monochloride in glacial acetic acid is also regioselective, giving the 3-iodo derivative **1g**.

Recently we reported regioselective monobromination of carprofen to form 3-bromocarprofen (Popa et al., 2019; Popa et al., 2023). This was achieved by reaction of carprofen with an equimolar amount of bromine in glacial acetic acid at 50-60 °C. Under similar reaction conditions and by using two moles of bromine, 3,8-dibromocarprofen **1h** was obtained by a regioselective double electrophilic substitution reaction (**Scheme 1**). 3,8-Dibromocarprofen was easily transformed into its methyl ester **1i** (**Scheme 1**) by esterification with methanol in the presence of H_2_SO_4_ at room temperature. The chemical structures were assigned based on NMR, IR spectroscopy and X-ray analysis.

Concerning compound **1a**, the presence of five aromatic protons in the H-NMR spectrum and their multiplicity indicate that monosubstitution takes place at the position 3 of the carbazole ring. The H-1 and H-4 protons appear as sharp singlets at 8.46 and 9.09 ppm. The two doublets that are observed result from a *meta* coupling between atoms H-7 and H-5. The most deshielded signal has a chemical shift of 12.1 ppm and corresponds to the proton of the NH group. The C-NMR spectrum presents all the signals as expected. The signal at 172.5 ppm is that with the highest shift in the NMR spectrum, corresponding to the carbon atom of the ester group. The relevant bands in the IR spectrum of **1a** include that at 1724 cm^-1^ corresponding to the carbonyl group and the one of the NH group appearing at 3329 cm^-1^.

The new *N*-substituted carprofen derivatives **1b-d** and **III** obtained by alkylation of the nitrogen atom were characterized by IR and NMR spectroscopy. The absence of both, the N-H signal from the H-NMR spectra and the IR bands for the NH group is good evidence for N-alkylation. The structures of the new carprofen halogenated esters **1e-g** were assigned by IR and NMR spectroscopy. In the H-NMR spectra of the esters **1e**and**1f** hydrogen atoms H-1 and H-4 appear as sharp singlets. The multiplicities of the protons of the benzene ring bearing the chlorine atom are the same in all three compounds. In the H-NMR spectrum of the ethyl ester **1f** it was observed that the methylene protons of the ethyl group appear as a multiplet instead of a quartet. The magnetic non-equivalence of these protons is due to the chiral carbon center in the molecule. The carbon NMR spectra present all the expected signals. The presence of bromine and iodine atoms at C-3 of the carbazole ring induces a significant shielding of this atom relative to the starting materials.

The structure of 3,8-dibromocarprofen was assigned on the basis of NMR data and confirmed by X-ray diffraction. The H-NMR spectrum of the aromatic region of **1h** exhibited the presence of four hydrogen atoms. The H-1 and H-4 protons appear as sharp singlets with chemical shifts of 7.56 ppm and 8.54 ppm whereas H-5 and H-7 are two doublets with a coupling constant of 1.8 Hz which indicated a *meta* coupling between the two protons, and as a consequence, the subsequent step of bromination of carprofen takes place at C-8. The NH proton is strongly deshielded and has the chemical shift at 11.66 ppm. The carbon NMR spectrum presents the expected signals, the main feature being that occurring at 175.2 ppm, attributed to the carbon of the carboxylic group. The bromination at C-8 induces a shielding of the C-8 from 112.2 ppm to 104.5 ppm. The presence of the NH group is confirmed in the IR spectrum by the vibrational band at 3348 cm^-1^ whereas the band corresponding to the C=O group appears at 1689 cm^-1^. The regioselective dibromination of carprofen was confirmed by single crystal X-ray diffraction.

### 3.2 Single crystal X-ray analyses of 1h and 1i

**Figure 2:**
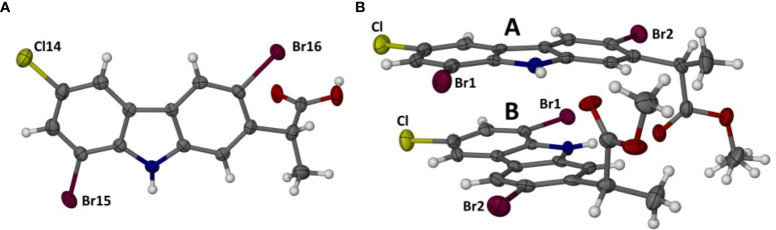
Perspective views of the single molecule in the ASU of compound 1h **(A)** and the two independent molecules (A, B) in the ASU of 1i **(B)**. Atoms are drawn as thermal ellipsoids at the 50% probability level. In molecule A, the methyl hydrogen atoms of the ester moiety were found to be disordered over two orientations, as indicated.

The new derivatives 3,8-dibromocarprofen (**1h**) and its methyl ester (**1i**) crystallize in the monoclinic space group *C*2/c and the triclinic space group *P* (–1) respectively. **Figure 2** shows the structures of their respective asymmetric units (ASUs), consisting of a single molecule of the free acid **1h** and two crystallographically independent molecules of the methyl ester **1i**. The molecules are brominated at the 3 and 8 positions. As expected, detailed analyses of the crystal structures of **1h** and **1i** revealed considerable differences in their modes of molecular association, as described below.

**Figure 3:**
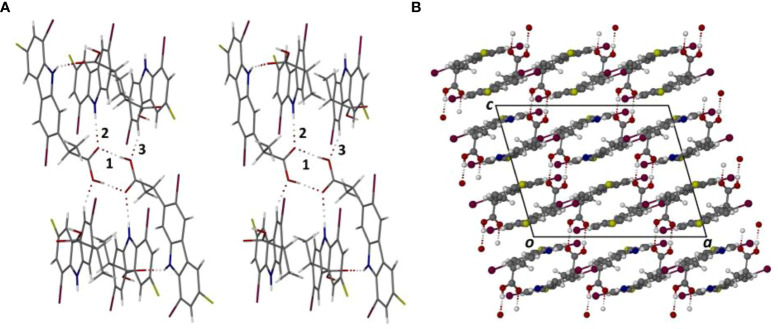
Stereoscopic view of the principal hydrogen bonding interactions in the crystal of 1h **(A)** and the resulting packing diagram viewed parallel to the crystal b-axis **(B)**.

The crystal structure of the free acid **1h** is based on centrosymmetric hydrogen-bonded carboxylic acid dimers that interlink with one another *via* additional hydrogen bonds. **Figure 3A** is a stereoscopic view of a representative dimer with nearest-neighboring molecules of **1h** appended to the dimer. Three unique hydrogen bonds are indicated, H-bond 1 and its inversion-related counterpart being those forming the well-known acid-acid dimer synthon with graph-set notation *R* ^2^_2_ (8), while intermolecular H-bond 2 links the carbonyl oxygen atom of the acid with the donor N-H group of a neighboring **1h** molecule, and H-bond 3 links the oxygen atom of the -OH group with a C-H donor group on another molecule of **1h**.

The geometrical parameters for these H-bonds (in order of decreasing strength) are as follows:

1. O20-H20···O19^i^ (i = 3/2-x,3/2-y,1-z), H···O 1.81 Å, O···O 2.650(4) Å, ∠O-H···O 175°;
2. N10-H10···O19^ii^(ii = 1-x,y,1/2-z), H···O 2.23Å, N···O 3.056(4)Å, ∠N-H···O 156°;
3. C4-H4···O20^iii^(iii = 3/2-x,-1/2+y,1/2-z), H···O 2.44Å, C···O 3.304(5) Å, ∠C-H···O 151°.

In addition to the hydrogen bonds that direct the overall supramolecular structure, π-stacking of the tricyclic moieties is very prominent. From **Figure 3A**, it is evident that there are two orientations of the otherwise nearly parallel carbazole moieties, leading to numerous, significantly short (< 3.5 Å) inter-ring centroid-to-centroid (Cg···Cg) distances that indicate strong π-interactions. The two unique π-interactions between the respective pyrrole rings, for example, feature Cg···Cg distances of only 3.349(2) and 3.404(2) Å. **Figure 3B**, the crystal packing viewed nearly parallel to the carbazole planes, shows the domains of π-interaction and their spatial arrangement more clearly. Given our previously mentioned interest in the occurrence of halogen bonding and the fact that each molecule of **1h** contains two bromine atoms and a chlorine atom, we expected that such interactions might also play a role in crystal cohesion. However, a careful inspection of interhalogen atom distances and halogen···O/N distances showed that there are no interactions of this type, evidently owing to the strong H-bonding and π-interactions that direct the supramolecular assembly.

**Figure 4:**
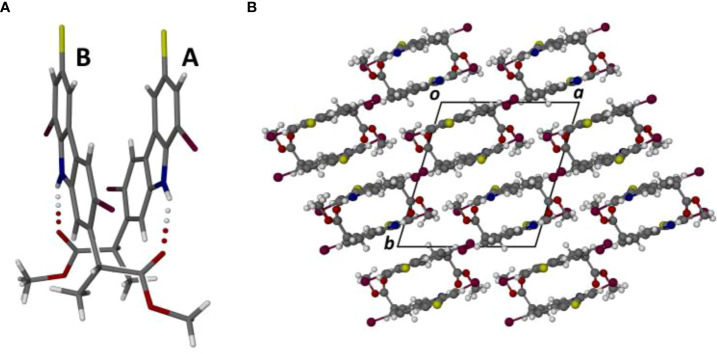
Perspective view of the dimeric motif formed by the association of molecules A and B in the crystal of compound 1i **(A)**, and the resulting packing diagram viewed parallel to the crystal c-axis **(B)**.

Methylation of the carboxylic acid group of **1h** results in the derivative **1i** which features a significantly different mode of molecular association in the crystal. **Figure 4A** shows the hydrogen bonded dimer formed between the independent molecules A and B in the ASU. The geometrical parameters for the two unique head-to-tail N-H···O hydrogen bonds are as follows:

1. N10A-H10A···O20B: H···O 2.24 Å, N···O 3.023(5) Å, ∠N-H···O 149°
2. N10B-H10B···O20A: H···O 2.19 Å, N···O 2.995(6) Å, ∠N-H···O 151°.

The dimeric structure, with pseudo-twofold rotational symmetry, is further stabilized by a strong π-interaction between the two pyrrole rings, for which the Cg···Cg distance is only 3.390 (3) Å; this is the minimum inter-ring distance in the crystal structure of **1i** and the only one < 3.50 Å. The dimeric units are linked pairwise by one C-H···O hydrogen bond and its inversion-related counterpart. **Figure 4B** shows the resulting packing arrangement in the crystal. A search for halogen bonding interactions yielded only one Type 1 contact C-Cl···Cl-C with Cl···Cl distance 3.438(2) Å.

Finally, we note that the supramolecular features described for the crystals of **1h** and **1i** resemble those identified recently in the crystal structures of both 3-bromocarprofen and 3-iodocarprofen and their respective methyl esters (Dumitrascu et al., 2022).

### 3.3 Drug-likeness, pharmacokinetic and pharmacogenomic profiles of compounds 1a-1i

The results generated from the medicinal chemistry filtering (Lipinski, Ghose, Veber, and Egan) are shown in **Table 1**.

**Table 1:** The drug-likeness features according to Lipinski, Veber, Ghose, and Egan rules.

Compound	Lipinski	Ghose	Veber	Egan	Bioavailability Score
**1a**	Yes	Yes	Yes	Yes	0.55
**1b**	Yes	Yes	Yes	Yes	0.55
**1c**	Yes; 1 violation: MLOGP>4.15	Yes	Yes	Yes	0.55
**1d**	Yes	Yes	Yes	Yes	0.55
**1e**	Yes; 1 violation: MLOGP>4.15	Yes	Yes	Yes	0.55
**1f**	Yes; 1 violation: MLOGP>4.15	Yes	Yes	Yes	0.55
**1g**	Yes; 1 violation: MLOGP>4.15	Yes	Yes	Yes	0.55
**1h**	Yes; 1 violation: MLOGP>4.15	No; 1 violation: WLOGP>5.6	Yes	Yes	0.85
**1i**	Yes; 1 violation: MLOGP>4.15	No; 1 violation: WLOGP>5.6	Yes	Yes	0.55

Our results show that compounds **1a-1i** comply with the drug-likeness rules, indicating that these compounds have a possible drug effect and good bioavailability. Even though for the compounds **1h** and **1i**, a log P greater than 5.6 was recorded and the Ghose filter does not apply to these compounds, we also included compounds **1h** and **1i** in our analysis considering that these compounds are in agreement with Lipinski, Veber and Egan filters.

Furthermore, the ADME-predictable properties of the compounds **1a-1i** were evaluated (**Table 2**). We evaluated the ADME characteristics, with emphasis on (i) intestinal absorption, (ii) blood-brain barrier (BBB), and (iii) P-glycoprotein inhibitor/substrate and (iv) a renal organic cation transporter 2 (OCT2) substrate. The results revealed that (i) all compounds exhibited good intestinal absorption and good BBB permeability; this indicates that all compounds may be prepared for oral administration. Regarding P-glycoprotein inhibitor/substrate the tested compounds are neither substrates nor inhibitors, except compound **1c**. Similar results were obtained also for the elimination rate, demonstrating that none of the compounds (excepting **1c**) are renal OCT2 substrates.

**Table 2:** Computational pharmacokinetic profiles for compounds 1a-1i.

Compounds	Human Intestinal Absorption	Blood-Brain Barrier	P-glycoprotein inhibitor/substrate	OCT2 inhibitor
**1a**	yes	yes	no/no	no
**1b**	yes	yes	no/no	no
**1c**	yes	yes	yes/yes	yes
**1d**	yes	yes	no/no	no
**1e**	yes	yes	no/no	no
**1f**	yes	yes	no/no	no
**1g**	yes	yes	no/no	no
**1h**	yes	yes	no/no	no
**1i**	yes	yes	no/no	no

The pharmacogenomic profiles of the compounds **1a-1i** for critical cytochromes, namely CYP3A4, CYP2C9, CYP2D6, CYP2C19 and CYP1a2, are presented in **Table 3**. The results regarding metabolic pathways of compounds **1a**-**1i**, showed that compounds **1a**-**1g** and **1i** presented affinities for CYP3A4 as activators but not as inhibitors, while **1h** seems not to interact with this cytochrome. Interesting results were obtained for metabolic pathways related to CYP2D6, suggesting that the compounds **1a**-**1i** are not playing the role of inhibitors or substrates, but instead all compounds inhibited CYP1a2. Detailed results are presented in **Table 3**.

**Table 3:** The inhibitor/substrate features of compounds 1a-1i at CYP2D6, CYP3A4, CYP1a2, CYP2C19, and CYP2C9.

Compounds	CYP3A4 substrate/inhibitors	CYP2C9 substrate/inhibitors	CYP2D6 substrate/inhibitors	CYP2C19 inhibition	CYP1a2 inhibition
**1a**	yes/no	no/yes	no/no	yes	yes
**1b**	yes/no	no/yes	no/no	yes	yes
**1c**	yes/no	no/yes	no/no	yes	yes
**1d**	yes/no	no/no	no/no	yes	yes
**1e**	yes/no	no/yes	no/no	yes	yes
**1f**	yes/no	no/yes	no/no	yes	yes
**1g**	yes/no	no/yes	no/no	yes	yes
**1h**	no/no	no/no	no/no	no	yes
**1i**	yes/yes	no/yes	no/no	yes	yes

In this study, great importance was given to the prediction of the toxicity of the compounds (**Table 4**). Very detailed predicted toxicity profiles for all compounds **1a**-**1i** are presented. Our results revealed that most of the tested compounds (i) are not carcinogenic, (ii) do not induce eye corrosion or irritation and (iii) do not induce nephrotoxicity. Regarding the mutagenic effect, only **1a** might induce AMES effects. All compounds **1a**-**1i** induced hepatic, respiratory and reproductive toxicity. Interesting results were obtained for the predicted mitochondrial toxicity profiles, with only **1c**, **1d** and **1f** possibly inducing mitochondrial toxicity.

**Table 4:** The predicted toxicity profiles of compounds 1a-1i.

Toxicity features	1a	1b	1c	1d	1e	1f	1g	1h	1i
Carcinogenicity	no	no	no	no	no	no	no	no	no
Eye corrosion /irritation	no	no	no	no	no	no	no	no	no
Ames	yes	no	no	no	no	no	no	no	no
Hepatotoxicity	yes	yes	yes	yes	yes	yes	yes	yes	yes
Respiratory toxicity	yes	yes	yes	yes	yes	yes	yes	yes	yes
Reproductive toxicity	yes	yes	yes	yes	yes	yes	yes	yes	yes
Mitochondrial toxicity	no	no	yes	yes	no	yes	no	no	no
Nephrotoxicity	no	no	no	no	no	no	yes	no	no
biodegradation	yes	no	no	no	no	no	no	no	no

### 3.4 Total antioxidant capacity measurements

The total antioxidant capacity was measured using the well-known DPPH assay and the results are presented in **Table 5**.

**Table 5:** TAC values (%) for compounds 1a-1i.

Compound	1a	1b	1c	1d	1e	1f	1g	1h	1i
TAC (%)	21	18	22	9	6	11	16	16	23

For compounds **1a-1i**, the TAC values showed that the highest antioxidant capacities were recorded for the compounds **1i > 1c > 1a** (~ 21-23%), while the lowest value was recorded for **1e** (6%). The most active compound **1i** bears one chlorine and two bromine atoms, as well as the CO_2_Me group. For the compounds **1a** and **1i**, the highest antioxidant activity can be correlated with the presence of the -NH group, which can facilitate the H atom transfer to DPPH.

### 3.5 Antimicrobial activity

The antimicrobial activity of the nine new derivatives has been assessed against planktonic and adhered bacteria. We have used four reference strains which are recommended by international standards to be used for assessing the antimicrobial activity of both current and novel antimicrobials. These reference strains have well known antibiotic susceptibility profiles, therefore the results obtained by different laboratories and in different studies could be easily compared and reproduced. The four strains are also representative for the Gram-positive (*S. aureus*, *E. faecalis*) and Gram-negative (*E. coli*, *P. aeruginosa*) species which are included in the lists of the most challenging antibiotic-resistant pathogens, such as ESKAPE, ESKAPE (E), ESCAPE, or the CDC list. These pathogens are requiring immediate focus for managing the underlying hospital and community-acquired infections, often associated with the development of bacterial biofilms and for fostering the discovery and development of efficient alternative therapeutic strategies.

In our study, the antimicrobial activity was evaluated through quantitative assays of MIC, MBC and MBEC, allowing to establish comparatively the efficiency of the tested compounds against planktonic and adherent Gram-positive and Gram-negative bacteria, as well as to gain insight regarding the bacteriostatic or bactericidal effect at the tested concentrations.

**Figure 5:**
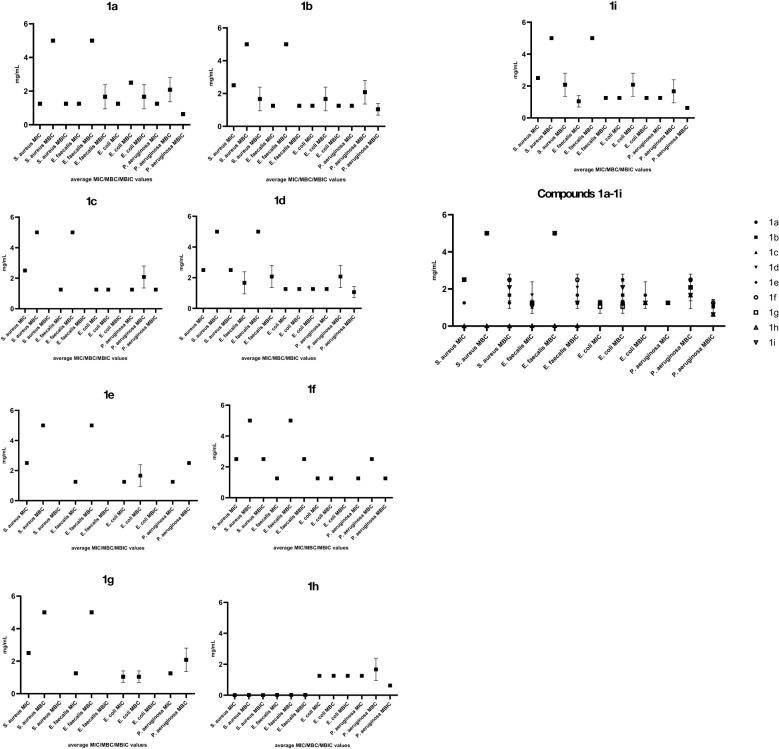
Graphic representation of the average MIC, MBC and MBEC values of the tested compounds, calculated from three replicates, for the four tested bacterial strains (Graph Prism 9.5.1, means with SD, N are represented, p<0.0001, two-way Anova, multiple comparisons).

Among the four tested strains, the Gram-negative ones, *P. aeruginosa* and *E*. *coli* strains proved to be more susceptible to all tested compounds, in comparison with the Gram-positive strains, both in planktonic and adherent growth state. From the two Gram-positive strains, *E. faecalis* proved to be more susceptible to the tested compounds in comparison with *S. aureus* (**Figure 5**). With few exceptions, the MIC and MBEC values were similar, while the MBC values were higher, suggesting a bacteriostatic activity of the tested compounds.

The compound **1h,** bearing two bromine atoms, has been by far the most active against all tested bacterial strains, with very low MIC values, especially against the two Gram-positive bacterial strains (0.090 -0.019 mg/mL). The MBC and MBEC values were very similar to the MIC values against the Gram-positive strains, suggesting the bactericidal effect of this compound as well as its anti-biofilm potential.

The compound **1a** bearing one chlorine atom, one nitro and one methoxycarbonyl group has also exhibited antibacterial and anti-biofilm activity against all four tested strains, the most significant inhibitory effect being noticed against *P. aeruginosa* and *S. aureus* strains. This compound has also exhibited a good antioxidant activity in the DPHH method. These results suggest that: i) the mechanism of the antibacterial activity of the new derivatives is not (exclusively) related to the generation of oxidative stress in the bacterial cells; ii) the potential antibacterial drug could exhibit also anti-inflammatory activity, thus reducing the severity of the clinical symptoms of bacterial infection as well as the possible side-effects of the drug. Further research is thus needed for elucidating the underlying antibacterial mechanisms and how the antioxidant activity of these compounds could influence their efficacy.

The compounds **1b, 1i, 1c** and **1d** proved to be active against *E. coli, P. aeruginosa* and *E. faecalis* strains, with MIC values ranging from 0.6 to 1.3 mg/mL, MBC values from 1.3 to 2.5 mg/mL and MBEC values from 0.6 to 1.3 mg/mL.

The lowest antibacterial activity was observed for **1e** and **1f** which inhibited the planktonic growth of *E. coli, P. aeruginosa* and *E. faecalis* strains (MIC of 1.3 mg/mL) and, in the case of **1f**, the biofilm growth of *P. aeruginosa* (MBEC of 1.3 mg/mL).

The results obtained in this paper suggest that the presence of one chlorine atom is associated with an extended antibacterial spectrum, while the presence of a bromine substituent increases the intensity of the observed antibacterial effect.

It also appears that the substitution of the heterocyclic nitrogen atom with a methyl group decreases the antibacterial activity of these derivatives.

## 4 Conclusions

In this paper, we report the preparation of a series of new carbazole derivatives based on the readily available anti-inflammatory drug carprofen by nitration, halogenation and N-alkylation of carprofen and its esters.

The structures of these carbazole compounds were assigned by NMR and IR spectroscopy. The regioselective electrophilic substitution at the carbazole ring was assigned from H NMR spectra. The single crystal X-ray structures of two representative derivatives obtained by dibromination of carprofen, namely 3,8-dibromocarprofen and its methyl ester, were also determined, revealing significant differences in their supramolecular assemblies.

The antimicrobial activity assays suggest that the compound **1h**, bearing one chlorine and two bromine atoms exhibited the most promising potential, especially against the Gram-positive strains, in planktonic and biofilm growth state. The compound **1a** (bearing one chlorine, one nitro and one CO_2_Me group) and **1i** (bearing one chlorine, two bromine atoms and a CO_2_Me group) exhibited the most intensive antibiofilm effect in the case of the *P. aeruginosa* strain and a good antioxidant potential. Moreover, these compounds comply with the drug-likeness rules, have good oral bioavailability and are not carcinogenic or mutagenic. Together, the obtained results demonstrate the potential of the new carbazole derivatives to be explored further for the development of novel antibacterial and antibiofilm agents.

## Data availability statement

The datasets presented in this study can be found in online repositories. The names of the repository/repositories and accession number(s) can be found in the article/supplementary material. CCDC 2242995 and 2242996 contain the supplementary crystallographic data for this paper. These data can be obtained from the CCDC, 12 Union Road, Cambridge CB2 1eZ, UK; Fax: +44-1223-336033; E-mail: deposit@ccdc.cam.ac.uk.

## Author contributions

FD, CL, M-CC, MRC, IZ, and SA contributed to conception and design of the study. DN and IV organized the database. MP and IV performed the statistical analysis, M-CC and MP performed the microbiology tests, IZ and PI performed the antioxidant tests, SA, AU, and CB performed *in silico* tests. FD and CL wrote the first draft of the manuscript. MRC, SA, IZ, MP, M-CC, and DN wrote sections of the manuscript. All authors contributed to the article and approved the submitted version.

## References

[B1] Addla D. Wen S.-Q. Gao W.-W. Maddili S. K. Zhang L. Zhou C.-H. (2016). Design, synthesis, and biological evaluation of novel carbazole aminothiazoles as potential DNA-targeting antimicrobial agents. MedChemComm 7, 1988–1994. doi: 10.1039/C6MD00357E

[B2] Arias C. A. Murray B. E. (2012). The rise of the Enterococcus: beyond vancomycin resistance. Nat. Rev. Microbiol. 10, 266–278. doi: 10.1038/nrmicro2761 22421879 PMC3621121

[B3] Avram S. Bologa C. Flonta M.-L. (2005). Quantitative structure-activity relationship by CoMFA for cyclic urea and nonpeptide-cyclic cyanoguanidine derivatives on wild type and mutant HIV-1 protease. J. Mol. Model. 11, 105–115. doi: 10.1007/s00894-004-0226-5 15714296

[B4] Avram S. Movileanu L. Mihailescu D. Flonta M.-L. (2002). Comparative study of some energetic and steric parameters of the wild type and mutants HIV-1 protease: a way to explain the viral resistance. J. Cell Mol. Med. 6, 251–260. doi: 10.1111/j.1582-4934.2002.tb00192.x 12169210 PMC6740297

[B5] Bandgar B. P. Adsul L. K. Chavan H. V. Jalde S. S. Shringare S. N. Shaikh R.. (2012). Synthesis, biological evaluation, and docking studies of 3-(substituted)-aryl-5-(9-methyl-3-carbazole)-1H-2-pyrazolines as potent anti-inflammatory and antioxidant agents. Bioorg Med. Chem. Lett. 22, 5839–5844. doi: 10.1016/j.bmcl.2012.07.080 22901385

[B6] Bem M. Baratoiu R. Radutiu C. Lete C. Mocanu S. Ionita G.. (2018). Synthesis and structural characterization of some novel methoxyamino derivatives with acid-base and redox behavior. J. Mol. Struct. 1173, 291–299. doi: 10.1016/j.molstruc.2018.06.114

[B7] Bordei Telehoiu A. T. Nuță D. C. Căproiu M. T. Dumitrascu F. Zarafu I. Ioniță P.. (2020). Design, synthesis and i*n* v*itro* characterization of novel antimicrobial agents based on 6-chloro-9H-carbazol derivatives and 1,3,4-oxadiazole scaffolds. Molecules 25. doi: 10.3390/molecules25020266 PMC7024163 31936505

[B8] Borger C. Brutting C. Julich-Gruner K. K. Hesse R. Kumar V. P. Kutz S. K.. (2017). Anti-tuberculosis activity and structure-activity relationships of oxygenated tricyclic carbazole alkaloids and synthetic derivatives. Bioorg Med. Chem. Lett. 25, 6167–6174. doi: 10.1016/j.bmc.2016.12.038 28094223

[B9] Buiu C. Putz M. V. Avram S. (2016). Learning the relationship between the primary structure of HIV envelope glycoproteins and neutralization activity of particular antibodies by using artificial neural networks. Int. J. Mol. Sci. 17. doi: 10.3390/ijms17101710 PMC5085742 27727189

[B10] Cascioferro S. Parrino B. Carbone D. Pecoraro C. Diana P. (2021). Novel strategies in the war against antibiotic resistance. Future Med. Chem. 13, 529–531. doi: 10.4155/fmc-2021-0009 33467930

[B11] Chan E. W. L. Yee Z. Y. Raja I. Yap J. K. Y. (2017). Synergistic effect of non-steroidal anti-inflammatory drugs (NSAIDs) on antibacterial activity of cefuroxime and chloramphenicol against methicillin-resistant Staphylococcus aureus. J. Glob. Antimicrob. Resist. 10, 70–74. doi: 10.1016/j.jgar.2017.03.012 28673701

[B12] Chang J. Lee R.-E. Lee W. (2020). A pursuit of Staphylococcus aureus continues: a role of persister cells. Arch. Pharm. Res. 43, 630–638. doi: 10.1007/s12272-020-01246-x 32627141

[B13] Chatterjee M. Anju C. P. Biswas L. Kumar V. A. Mohan C. G. Biswas R. (2016). Antibiotic resistance in Pseudomonas aeruginosa and alternative therapeutic options. Int. J. Med. Microbiol. 306, 48–58. doi: 10.1016/j.ijmm.2015.11.004 26687205

[B14] Cuong N. M. Wilhelm H. Porzel A. Arnold N. Wessjohann L. (2008). 1-O-substituted derivatives of murrayafoline A and their antifungal properties. Nat. Prod Res. 22, 950–954. doi: 10.1080/14786410701650212 18629709

[B15] Dabrovolskas K. Jonuskiene I. Sutkuviene S. Gudeika D. (2020). Synthesis and evaluation of antibacterial and antioxidative activities of carbazole derivatives. Chemija 31, 42–51. doi: 10.6001/chemija.v31i1.4170

[B16] Dineshkumar B. Mitra A. Mahadevappa M. (2011). Antidiabetic and hypolipidemic effects of mahanimbine (carbazole alkaloid) from Murraya koenigii (Rutaceae) Leaves. Int. J. Phytomed. 2, 22–30. doi: 10.5138/ijpm.2010.0975.0185.02004

[B17] Dumitrascu F. Udrea A.-M. Caira M. R. Nuta D. C. Limban C. Chifiriuc M. C.. (2022). In silico and experimental investigation of the biological potential of some recently developed carprofen derivatives. Molecules 27, 2722. doi: 10.3390/molecules27092722 35566083 PMC9101252

[B18] Eun Y.-J. Foss M. H. Kiekebusch D. Pauw D. A. Westler W. M. Thanbichler M.. (2012). DCAP: A broad-spectrum antibiotic that targets the cytoplasmic membrane of bacteria. J. Am. Chem. Soc. 134, 11322–11325. doi: 10.1021/ja302542j 22741745 PMC3516701

[B19] Ewers C. Gopel L. Prenger-Berninghoff E. Semmler T. Kerner K. Bauerfeind R. (2022). Occurrence of mcr-1 and mcr-2 colistin resistance genes in porcine Escherichia coli isolates (2010-2020) and genomic characterization of mcr-2-positive E. coli. Front. Microbiol. 13. doi: 10.3389/fmicb.2022.1076315 PMC9780603 36569100

[B20] Favia A. D. Habrant D. Scarpelli R. Migliore M. Albani C. Bertozzi S. M.. (2012). Identification and characterization of carprofen as a multitarget fatty acid amide hydrolase/cyclooxygenase inhibitor. J. Med. Chem. 55, 8807–8826. doi: 10.1021/jm3011146 23043222 PMC3764917

[B21] Fisher R. A. Gollan B. Helaine S. (2017). Persistent bacterial infections and persister cells. Nat. Rev. Microbiol. 15, 453–464. doi: 10.1038/nrmicro.2017.42 28529326

[B22] Foxman B. (2010). The epidemiology of urinary tract infection. Nat. Rev. Urol 7, 653–660. doi: 10.1038/nrurol.2010.190 21139641

[B23] Grande F. Bartolo A. Occhiuzzi M. Caruso A. Rocca C. Pasqua T.. (2021). Carbazole and simplified derivatives: novel tools toward β-adrenergic receptors targeting. Appl. Sci. 11, 5486. doi: 10.3390/app11125486

[B24] Guillonneau C. Nault A. Raimbaud E. Leonce S. Kraus-Berthier L. Pierre A.. (2005). Cytotoxic and antitumoral properties in a series of new, ring D modified, olivacine analogues. Bioorg. Med. Chem. Lett. 13, 175–184. doi: 10.1016/j.bmc.2004.09.047 15582462

[B25] Guo Y. Song G. Sun M. Wang J. Wang Y. (2020). Prevalence and therapies of antibiotic-resistance in staphylococcus aureus. Front. Cell Infect. Microbiol. 10. doi: 10.3389/fcimb.2020.00107 PMC7089872 32257966

[B26] Hieda Y. Anraku M. Choshi T. Tomida H. Fujioka H. Hatae N.. (2014). Antioxidant effects of the highly-substituted carbazole alkaloids and their related carbazoles. Bioorg Med. Chem. Lett. 24, 3530–3533. doi: 10.1016/j.bmcl.2014.05.050 24928405

[B27] Humphries R. M. Kelesidis T. Tewhey R. Rose W. E. Schork N. Nizet V.. (2012). Genotypic and phenotypic evaluation of the evolution of high-level daptomycin nonsusceptibility in vancomycin-resistant enterococcus faecium. Antimicrob. Agents Chemother. 56, 6051–6053. doi: 10.1128/AAC.01318-12 22948885 PMC3486580

[B28] Is O. D. Koyuncu F. B. Koyuncu S. Ozdemir E. (2010). A new imine coupled pyrrole–carbazole–pyrrole polymer: Electro-optical properties and electrochromism. Polymer (Guildf) 51, 1663–1669. doi: 10.1016/j.polymer.2010.02.020

[B29] Kaplancıklı Z. (2014). Synthesis of some novel Carbazole derivatives and evaluation of their antimicrobial activity. Marmara Pharm. J. 15, 105–109. doi: 10.12991/mpj.57879

[B30] Laborda P. Hernando-Amado S. Martínez J. L. Sanz-García F. (2022). “Antibiotic resistance in pseudomonas BT - pseudomonas aeruginosa,” in Biology, pathogenesis and control strategies. Eds. Filloux A. Ramos J.-L. (Cham: Springer International Publishing), 117–143. doi: 10.1007/978-3-031-08491-1_5

[B31] Lazaris A. Coleman D. C. Kearns A. M. Pichon B. Kinnevey P. M. Earls M. R.. (2017). Novel multiresistance cfr plasmids in linezolid-resistant methicillin-resistant Staphylococcus epidermidis and vancomycin-resistant Enterococcus faecium (VRE) from a hospital outbreak: co-location of cfr and optrA in VRE. J. Antimicrob. Chemother. 72, 3252–3257. doi: 10.1093/jac/dkx292 28961986

[B32] Lee C.-H. Su L.-H. Liu J.-W. Chang C.-C. Chen R.-F. Yang K.-D. (2014). Aspirin enhances opsonophagocytosis and is associated to a lower risk for Klebsiella pneumoniaeinvasive syndrome. BMC Infect. Dis. 14, 47. doi: 10.1186/1471-2334-14-47 24476545 PMC3916306

[B33] Lewis K. (2008). Multidrug Tolerance of Biofilms and Persister Cells. In: Romeo, T. (eds) Bacterial Biofilms. Springer, Berlin, Heidelberg. Current Topics in Microbio. Immunol. 322, 107–131. doi: 10.1007/978-3-540-75418-3_6 18453274

[B34] Limban C. Chifiriuc M. C. Caproiu M. T. Dumitrascu F. Ferbinteanu M. Pintilie L.. (2020). New substituted benzoylthiourea derivatives: from design to antimicrobial applications. Molecules 25. doi: 10.3390/molecules25071478 PMC7180980 32218209

[B35] Linden P. K. (2002). Treatment options for vancomycin-resistant enterococcal infections. Drugs 62, 425–441. doi: 10.2165/00003495-200262030-00002 11827558

[B36] Liu X. Zhao M. Fan X. Fu Y. (2021). Reshaping the active pocket of esterase Est816 for resolution of economically important racemates. Catal Sci. Technol. 11, 6126–6133. doi: 10.1039/D1CY01028J

[B37] Maitra A. Evangelopoulos D. Chrzastek A. Martin L. T. Hanrath A. Chapman E.. (2020). Carprofen elicits pleiotropic mechanisms of bactericidal action with the potential to reverse antimicrobial drug resistance in tuberculosis. J. Antimicrob. Chemother. 75, 3194–3201. doi: 10.1093/jac/dkaa307 32790867 PMC7566368

[B38] Malekian N. Agrawal A. A. Berendonk T. U. Al-Fatlawi A. Schroeder M. (2022). A genome-wide scan of wastewater E. coli for genes under positive selection: focusing on mechanisms of antibiotic resistance. Sci. Rep. 12. doi: 10.1038/s41598-022-11432-0 PMC9110714 35577863

[B39] Marinas I. C. Oprea E. Chifiriuc M. C. Badea I. A. Buleandra M. Lazar V. (2015). Chemical composition and antipathogenic activity of artemisia annua essential oil from romania. Chem. Biodivers 12, 1554–1564. doi: 10.1002/cbdv.201400340 26460560

[B40] Martin A. E. Prasad K. J. R. (2006). Synthesis and characterization of carbazole derivatives and their antimicrobial studies. Acta Pharm. 56, 79–86. 16613737

[B41] Martinez N. Luque R. Milani C. Ventura M. Banuelos Margolles A. (2018). A Gene Homologous to rRNA Methylase Genes Confers Erythromycin and Clindamycin Resistance in Bifidobacterium breve. Appl. Environ. Microbiol. 84, e02888–e02817. doi: 10.1128/AEM.02888-17 29500262 PMC5930361

[B42] Michiels J. E. Van den Bergh B. Verstraeten N. Michiels J. (2016). Molecular mechanisms and clinical implications of bacterial persistence. Drug Resist. Update 29, 76–89. doi: 10.1016/j.drup.2016.10.002 27912845

[B43] Miller C. Kong J. Tran T. T. Arias C. A. Saxer G. Shamoo Y. (2013). Adaptation of Enterococcus faecalis to daptomycin reveals an ordered progression to resistance. Antimicrob. Agents Chemother. 57, 5373–5383. doi: 10.1128/AAC.01473-13 23959318 PMC3811304

[B44] Naik N. Kumar H. V. Swetha H. (2010). Synthesis and evaluation of novel carbazole derivatives as free radical scavengers. Bulg Chem. Commun. 42, 40–45.

[B45] Olar R. Badea M. Chifiriuc M. C. (2022). Metal complexes-A promising approach to target biofilm associated infections. Molecules 27. doi: 10.3390/molecules27030758 PMC8838073 35164021

[B46] Pang Z. Raudonis R. Glick B. R. Lin T. J. Cheng Z. Y. (2019). Antibiotic resistance in Pseudomonas aeruginosa: mechanisms and alternative therapeutic strategies. Biotechnol. Adv. 37, 177–192. doi: 10.1016/j.biotechadv.2018.11.013 30500353

[B47] Paun A. Zarafu I. Caproiu M. T. Draghici C. Maganu M. Cotar A. I.. (2013). Synthesis and microbiological evaluation of several benzocaine derivatives. Comptes Rendus Chim. 16, 665–671. doi: 10.1016/j.crci.2013.03.012

[B48] Popa M. M. Man I. C. Draghici C. Shova S. Caira M. R. Dumitrascu F.. (2019). Halogen bonding in 5-iodo-1-arylpyrazoles investigated in the solid state and predicted bysolution 13C-NMR spectroscopy. CrystEngComm. 21, 7085–7093. doi: 10.1039/C9CE01263J

[B49] Popa M. M. Shova S. Dascalu M. Caira M. R. Dumitrascu F. (2023). Crystal structures of5-bromo-1-arylpyrazoles and their halogen bonding features. CrystEngComm 25, 86–94. doi: 10.1039/D2CE01355J

[B50] Remes C. Paun A. Zarafu I. Tudose M. Caproiu M. T. Ionita G.. (2012). Chemical and biological evaluation of some new antipyrine derivatives with particular properties. Bioorg. Chem. 41–42, 6–12. doi: 10.1016/j.bioorg.2011.12.003 22257969

[B51] Ruoff K. L. de la M L. Murtagh M. J. Spargo J. D. Ferraro M. J. (1990). Species identities of enterococci isolated from clinical specimens. J. Clin. Microbiol. 28, 435–437. doi: 10.1128/jcm.28.3.435-437.1990 2108992 PMC269638

[B52] Salih N. Salimon J. Yousif E. (2016). Synthesis and antimicrobial activities of 9H-carbazole derivatives. Arab J. Chem. 9, S781–S786. doi: 10.1016/j.arabjc.2011.08.013

[B53] Schulte R. H. Munson E. (2019). Staphylococcus aureus resistance patterns in wisconsin: 2018 surveillance of wisconsin organisms for trends in antimicrobial resistance and epidemiology (SWOTARE) program report. Clin. Med. Res. 17, 72–81. doi: 10.3121/cmr.2019.1503 31582419 PMC6886895

[B54] Sharma D. Kumar N. Pathak D. (2014). Synthesis, Characterization and biological evaluation of some newer carbazole derivatives. J. Serbian Chem. Soc. 79, 125–132. doi: 10.2298/JSC130123069S

[B55] Sharma G. Rao S. Bansal A. Dang S. Gupta S. Gabrani R. (2014). Pseudomonas aeruginosa biofilm: Potential therapeutic targets. BIOLOGICALS 42, 1–7. doi: 10.1016/j.biologicals.2013.11.001 24309094

[B56] Shirin H. Moss S. F. Kancherla S. Kancherla K. Holt P. R. Weinstein I. B.. (2006). Non-steroidal anti-inflammatory drugs have bacteriostatic and bactericidal activity against Helicobacter pylori. J. Gastroenterol. Hepatol. 21, 1388–1393. doi: 10.1111/j.1440-1746.2006.04194.x 16911681

[B57] Sivick K. E. Mobley H. L. (2010). Waging War against Uropathogenic Escherichia coli: Winning Back the Urinary Tract. Infect. Immun. 78, 568–585. doi: 10.1128/IAI.01000-09 19917708 PMC2812207

[B58] Srinivas K. Kumar C. R. Reddy M. A. Bhanuprakash K. Rao V. J. Giribabu L. (2011). D-π-A organic dyes with carbazole as donor for dye-sensitized solar cells. Synth Met. 161, 96–105. doi: 10.1016/j.synthmet.2010.11.004

[B59] Subashchandrabose S. Mobley H. L. T. (2017). Virulence and fitness determinants of uropathogenic escherichia coli. Urinary Tract Infections, 235–261. doi: 10.1128/9781555817404.ch12 PMC4566162 26350328

[B60] Tansirichaiya S. Moyo S. J. Al-Haroni M. Roberts A. P. (2021). Capture of a novel, antibiotic resistance encoding, mobile genetic element from Escherichia coli using a new entrapment vector. J. Appl. Microbiol. 130, 832–842. doi: 10.1111/jam.14837 32881179

[B61] Udrea A. M. Gradisteanu Pircalabioru G. Boboc A. A. Mares C. Dinache A. Mernea M.. (2021). Advanced bioinformatics tools in the pharmacokinetic profiles of natural and synthetic compounds with anti-diabetic activity. Biomolecules 11. doi: 10.3390/biom11111692 PMC8615418 34827690

[B62] Udrea A.-M. Mernea M. Buiu C. Avram S. (2020). Scutellaria baicalensis Flavones as Potent Drugs against Acute Respiratory Injury during SARS-CoV-2 Infection: Structural Biology Approaches. Processes 8. doi: 10.3390/pr8111468

[B63] Udrea A. M. Puia A. Shaposhnikov S. Avram S. (2018). Computational approaches of new perspectives in the treatment of depression during pregnancy. Farmacia 66, 680–687. doi: 10.31925/farmacia.2018.4.18

[B64] Venkateswaran P. Lakshmanan P. M. Muthukrishnan S. Bhagavathi H. Vasudevan S. Neelakantan P.. (2022). Hidden agenda of Enterococcus faecalis lifestyle transition: planktonic to sessile state. Future Microbiol. 17, 1051–1069. doi: 10.2217/fmb-2021-0212 35899477

[B65] Vestergaard M. Frees D. Ingmer H. (2019). Antibiotic resistance and the MRSA problem. Microbiol. Spectr. 7, 7.2.18. doi: 10.1128/microbiolspec.GPP3-0057-2018 PMC11590431 30900543

[B66] Vlad I. M. Nuta D. C. Chirita C. Caproiu M. T. Draghici C. Dumitrascu F.. (2020). In silico and i*n* v*itro* experimental studies of new dibenz[b,e]oxepin-11(6H)one O-(arylcarbamoyl)-oximes designed as potential antimicrobial agents. Molecules 25. doi: 10.3390/molecules25020321 PMC7024208 31941125

[B67] Vlaicu I. D. Olar R. Maxim C. Chifiriuc M. C. Bleotu C. Stănică N.. (2019). Evaluating the biological potential of some new cobalt (II) complexes with acrylate and benzimidazole derivatives. Appl. Organomet. Chem. 33, e4976. doi: 10.1002/aoc.4976

[B68] Wu S. C. Liu F. Zhu K. Shen J. Z. (2019). Natural products that target virulence factors in antibiotic-resistant staphylococcus aureus. J. Agric. Food Chem. 67, 13195–13211. doi: 10.1021/acs.jafc.9b05595 31702908

[B69] Xue Y.-J. Li M.-Y. Jin X.-J. Zheng C.-J. Piao H.-R. (2021). Design, synthesis and evaluation of carbazole derivatives as potential antimicrobial agents. J. Enzyme Inhib Med. Chem. 36, 295–306. doi: 10.1080/14756366.2020.1850713 33404277 PMC7801072

[B70] Yang H. Lou C. Sun L. Li J. Cai Y. Wang Z.. (2019). admetSAR 2.0: web-service for prediction and optimization of chemical ADMET properties. Bioinformatics 35, 1067–1069. doi: 10.1093/bioinformatics/bty707 30165565

[B71] Yimer E. M. Mohammed O. A. Mohammedseid S. I. (2018). Pharmacological exploitation of non-steroidal anti-inflammatory drugs as potential sources of novel antibacterial agents. Anti-Infective Agents 17, 81–92. doi: 10.2174/2211352516666181008114542

[B72] Yin Z. Wang Y. Whittell L. R. Jergic S. Liu M. Harry E.. (2014). DNA replication is the target for the antibacterial effects of nonsteroidal anti-inflammatory drugs. Chem. Biol. 21, 481–487. doi: 10.1016/j.chembiol.2014.02.009 24631121

[B73] Zhang Y. Tangadanchu V. K. R. Cheng Y. Yang R.-G. Lin J.-M. Zhou C.-H. (2018). Potential antimicrobial isopropanol-conjugated carbazole azoles as dual targeting inhibitors of enterococcus faecalis. ACS Med. Chem. Lett. 9, 244–249. doi: 10.1021/acsmedchemlett.7b00514 29541368 PMC5846035

